# Metabolic phenotyping of pilomotor seizures in autoimmune encephalitis

**DOI:** 10.1111/cns.14192

**Published:** 2023-03-27

**Authors:** Yueqian Sun, Xiaobin Zhao, Lin Ai, Qun Wang

**Affiliations:** ^1^ Department of Neurology Beijing Tiantan Hospital, Capital Medical University Beijing China; ^2^ Department of Nuclear Medicine Beijing Tiantan Hospital, Capital Medical University Beijing China; ^3^ China National Clinical Research Center for Neurological Diseases Beijing China; ^4^ Beijing Institute of Brain Disorders, Collaborative Innovation Center for Brain Disorders Capital Medical University Beijing China

**Keywords:** autoimmune encephalitis, FDG‐PET, pathological mechanism, pilomotor seizures

## Abstract

**Objectives:**

Ictal piloerection (IP) is an uncommon symptom in focal epilepsy and is associated with autoimmune encephalitis (AE). However, the networks involved in AE‐associated IP are still unclear. To have a better understanding of IP underlying mechanisms, the current study investigated whole‐brain metabolic networks for the analysis of AE‐associated IP.

**Methods:**

Patients with AE and IP diagnosed at our Institute between 2018 and 2022 were selected. We then investigated the brain regions associated with AE‐associated IP using positron emission tomography (PET). Anatomometabolic changes (interictal ^18^F fluorodeoxyglucose PET) in AE patients with IP were compared with those of AE patients of similar age without IP (*p*‐voxel <0.001, uncorrected).

**Results:**

Sixteen patients showed significant IP. The overall IP prevalence was 4.09% of patients with AE and 12.9% of patients with limbic encephalitis. The most common autoantibodies were against LGI1 (68.8%) followed by GAD65 (6.3%), NMDA (6.3%), GABAb (6.3%), CASPR2 (6.3%), and antibodies recognizing both GAD65 and mGLUR5 (6.3%). Most patients responded well to immunotherapy. Analysis of the imaging results at the voxel level showed that patients with IP had hypermetabolic changes in the right inferior temporal gyrus, suggesting involvement of this brain region in IP.

**Conclusions:**

Our findings indicate that IP as an uncommon AE‐associated manifestations should be recognized. We observed that the metabolic pattern of IP was conspicuous in the right inferior temporal gyrus.

## Graphical abstract

Significant cluster of brain ^18^F‐FDG‐PET hypermetabolism was found at the group level for the patients with ictal piloerection. In comparison to patients without ictal piloerection, patients with ictal piloerection presented with significant brain hypermetabolism of the right inferior temporal gyrus (*p*‐voxel <0.001, uncorrected). Results are expressed as *T*‐score.

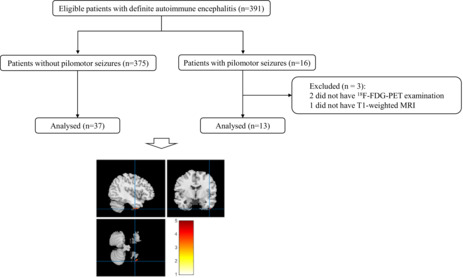

## 1 INTRODUCTION

Autoimmune encephalitis (AE) is typified by inflammation and autoantibodies against various proteins expressed on the surfaces of neurons, synaptic antigens, or intracellular proteins.^1^ Symptoms include psychiatric and memory disturbances, autonomic dysregulation, and seizures.^2^, ^3^, ^4^ The most common autoantibodies are directed against N‐methyl‐D‐aspartate receptor (NMDAR), leucine‐rich glioma‐inactivated protein 1 (LGI1), contactin‐associated protein‐like 2 (CASPR2), and γ‐aminobutyric acid B receptor (GABABR).^5^ Limbic *encephalitis (LE) primarily affects the medial temporal lobes and limbic regions such as the orbital cortex, cingulate gyrus, and hypothalamus* ^6^ with symptoms including psychiatric and short‐term memory disturbances, as well as seizures. The most common autoantibodies found in LE are against LGI1, CASPR2, GABABR, and glutamic acid decarboxylase 65 (GAD65).^7^ Anti‐LGI1 antibodies are characteristic of limbic autoimmune encephalitis (LGI1‐autoantibody‐associated LE (LGI1‐LE)),^8^ which is associated with facio‐brachial dystonic seizures (FBDS) that occur briefly and multiple times each day^9^ although other seizure types, specifically, motor, gelastic, and focal autonomic seizures, as well as impaired awareness, also occur. It is important to recognize these subtle types of seizure to diagnose and treat the underlying cause.

Ictal piloerection (IP) has been observed in LE, among other neurological disorders, and is usually classified as an autonomic epilepsy.^10^, ^11^ It is an uncommon accompaniment of focal seizures, with the result that the epilepsy diagnosis may be missed. It can occur on either one or both sides of the body and is often associated with autonomic manifestations such as sweating, chills, or pallor.^12^ Although IP is thought to be related to temperature dysregulation, its cause remains unknown. Piloerection has been associated with stimulation of various brain regions, including the hippocampus, insula, midbrain, amygdala, and medial prefrontal cortex, suggesting that ictal IP may be linked to autonomic control.^13^ The use of network approaches in the investigation of the pathophysiology of LE‐associated IP may provide insight into the etiology, diagnosis, and management of this disease.

^18^F‐fluoro‐2‐deoxy‐d‐glucose positron emission tomography (^18^F‐FDG‐PET) enables in vivo imaging and examination of brain function, and has been shown capable of diagnosing AE.^14^ Quantitative analysis of ^18^F‐FDG‐PET data can also be performed and have proved advantageous in the evaluation of a variety of neurological disorders.^15^ However, the metabolic activity associated with AE‐associated IP is unknown and the neural mechanisms underlying it remain obscure. The current study, therefore, investigated whole‐brain metabolic networks for the analysis of AE‐associated IP.

## 2 METHODS

### 2.1 Study participants

The study was approved by the institutional review board of Beijing Tiantan Hospital and written informed consent was obtained from all participants.

The data of patients diagnosed with AE between December 2018 and October 2022 in Beijing Tiantan Hospital were retrospectively evaluated for the incidence of IP. Patients had been diagnosed with AE in accordance with accepted criteria updated in 2016.^16^ Cases with IP as a major ictal characteristic were identified.

### 2.2 EEG and imaging

Conventional long‐term video‐electroencephalogram (EEG) recording was performed on each patient with standard 10–20 system electrodes. Magnetic resonance imaging (MRI) was performed with a 3T MRI system (Signa HD xt *3 T* Volume, *GE*, *GE* Healthcare, USA) while ^18^F‐FDG‐PET scans were done using a PET/CT scanner (acquisition parameters *see below*).

### 2.3 Functional assessment and evaluation of outcomes

For most patients, the Montreal Cognitive Assessment (MOCA) and the Mini‐Mental State Examination (MMSE) were used at the disease peak for assessing cognition. Subjects were assessed *for seizure* occurrence both at the disease peak and at follow‐up.

### 2.4 Neuroimaging data acquisition

Patients without ^18^F‐FDG‐PET images, poor quality images, or no MRI information were excluded. The patients received the same neuroimaging under resting conditions using a GE Discovery ST PET‐CT system (field of view, 300 mm; matrix, 192 × 192; slice thickness, 3.27 mm). Patients were given an intravenous injection of ^18^F‐FDG (310 MBq/70 kg body weight) and rested in a darkened room for 40 min. The ordered subset expectation maximization (OSEM) algorithm (16 iterations and six subsets with a 5‐mm Gaussian post filter) was used for reconstruction of the PET data. Normalization of images used germanium‐generated transmission scans.

### 2.5 Whole‐brain metabolic pattern analysis

**FIGURE 1:**
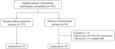
Flowchart of patient selection. ^18^F‐FDG‐PET, ^18^F‐fluorodeoxy‐glucose positron emission tomography.

Data analysis was done with statistical parametric mapping 12 (SPM12) software (Wellcome Department of Cognitive Neurology, University College, London, UK) running on Matlab 2021a (MathWorks Inc.). The ^18^F‐FDG‐PET images were initially linearly co‐registered with the individual T1WI MRI and the co‐registration was checked visually in terms of specific anatomical structures (specifically, the scalp and lateral ventricles) and low metabolic levels in the cerebrospinal fluid. The images were then normalized against the Montreal Neurological Institute atlas using a 12‐parameter affine transformation, followed by nonlinear transformations and a trilinear interpolation, resulting in images of 2 × 2 × 2 mm voxels. A Gaussian filter (8‐mm full‐width half‐maximum) was then used to smooth out variations in the gyral structures to enhance the signal‐to‐noise ratio. Proportional scaling was used to decrease individual variations in intensity. Group comparisons were used to assess metabolic patterns using whole‐brain metabolic voxel‐wise independent two‐sample *t*‐tests, using age and sex as nuisance variables: patients with IP versus patients without IP (*n* = 37). It should be mentioned that as all participants with IP included in the voxel‐wise analysis of ^18^F‐FDG‐PET have confirmed LE diagnoses, we selected LE patients as controls (Figure 1). Metabolic differences between AE patients with IP and those without IP were evaluated at the whole‐brain voxel‐based level using an uncorrected height threshold (voxel‐level significance) of *p* < 0.001 (corresponding to a *T*‐value of 3.42). Lastly, the xjView SPM extension (Cui & Li, Human Neuroimaging Lab, Baylor College of Medicine) was used for visualization of the anatomical locations of the MNI peaks of significantly different clusters.

## 3 RESULTS

### 3.1 Patient characteristics

A total of 391 definite hospitalized AE cases were enrolled in the study. Overall, we retrospectively screened 256 patients with NMDA‐autoantibody‐associated AE, 76 with LGI1‐LE, 21 with GABAb‐autoantibody‐associated LE (GABAb‐LE), 13 with GAD‐autoantibody‐associated LE (GAD‐LE), 6 with CASPR2‐autoantibody‐associated LE (CASPR2‐LE), and 4 with metabotropic glutamate receptor 5‐autoantibody‐associated AE (mGLUR5‐AE), as well as patients with anti‐amphiphysin (*n* = 7), anti‐Hu (*n* = 3), ‐Yo (*n* = 2), ‐CV2 (*n* = 2), and anti‐AMPAR encephalitis (*n* = 1). We finally identified 16 patients with IP (prevalence 4.09%, 8 males, mean age = 46.7 years, age range = 21–83 years; information presented in Table 1). The average epilepsy duration was 12.2 months (range 0.3–78). All patients had temporal lobe epilepsy (TLE). Eleven had LGI1, one anti‐NMDA, one anti‐CASPR2, one GABAb, one anti‐GAD65, and another was double‐positive for anti‐GAD65 and anti‐mGLUR5 autoantibodies (Table 1).

**TABLE 1:** Clinical features of 16 patients analyzed.

Patient	Sex/Age	Language domination	Antibody	Duration of disease (m)	Cognitive deficits (MMSE/MOCA)	MRI lesion	EEG (interictal/ictal)
1	M/57	L	LGI1	8	Yes (25/21)	Bilateral hippocampal atrophy	Slow waves and sharp waves in the bilateral frontal and anterior middle temporal areas‐ T ictal activity
2	M/64	L	LGI1	2	Yes (25/23)	Normal	Occasional sharp waves at the left sphenoidal electrode/NA
3	F/21	L	LGI1	1	Yes (21/19)	Bilateral hippocampal atrophy	Slow waves and sharp waves in the bilateral frontal and anterior temporal areas/T ictal activity
4	F/65	L	LGI1	0.5	Yes (20/11)	High FLAIR signal on left hippocampus and parahippocampal gyrus	Slow waves in the bilateral frontal and temporal areas; T ictal activity
5	F/30	L	LGI1	18	Yes (27/21)	High FLAIR signal and atrophy on left hippocampus; right hippocampus and amygdala were swollen	Normal/NA
6	M/58	L	LGI1	24	No (30/25)	Normal	Sharp slow waves in the left frontal and anterior middle temporal areas/NA
7	F/40	L	LGI1	4	No (28/28)	Normal	Sharp and slow waves in the left anterior middle temporal areas/NA
8	M/45	L	LGI1	6	No (NA)	High FLAIR signal and atrophy on left hippocampus; right hippocampus and amygdala were swollen	Normal/NA
9	M/83	L	LGI1	1.5	Yes (NA)	Bilateral hippocampal atrophy	Slow waves in the bilateral frontotemporal area/NA
10	M/60	L	LGI1	0.3	No (NA)	Normal	Slow waves and sharp waves in the left temporal area/left theta rhythmic activity, temporal ant
11	F/61	L	LGI1	3	Yes (29/23)	High FLAIR signal and swell on right hippocampus	Slow waves in the bilateral frontal and anterior middle temporal areas/T ictal activity
12	F/23	L	NMDA	2	Yes (NA)	Normal	Slow waves and sharp waves in the right temporal areas/right theta rhythmic activity, temporal ant
13	F/35	L	GAD65	78	No (29/26)	Normal	Sharp waves in the left anterior temporal areas/T ictal activity
14	F/26	L	GAD & mGLUR5	17	Yes (30/23)	High FLAIR signal on bilateral hippocampus and amygdala; bilateral hippocampal atrophy	Slow waves and sharp waves in the bilateral frontal and anterior middle temporal areas/Temporal ant
15	M/32	L	CASPR2	18	Yes (20/11)	Abnormal signals were observed in bilateral hippocampus and amygdalas	Normal/NA
16	M/47	L	GABAb	12	Yes (NA)	Normal	Normal/Theta rhythmic activity in left frontal and anterior middle temporal areas

Abbreviations: Ant, anterior; CASPR2, contactin‐associated protein‐like 2; F, female; GABAb, γ‐aminobutyric acid B; GAD65, glutamic acid decarboxylase 65; L, left; LGI1, leucine‐rich glioma‐inactivated protein 1; M, male; mGLUR5, metabotropic glutamate receptor 5; MMSE, the Mini‐Mental State Examination; MOCA, Montreal Cognitive Assessment; m, months; NA, not available; NMDA, N‐methyl‐D‐aspartate; T, temporal.

The frequency of pilomotor seizures varied among patients, from once every few days to over tens of times per day. In some patients, the seizure frequency increased significantly with emotional changes such as nervousness, anxiety, and agitation. Apart from patient #1, IP was observed in the head‐thoracic part of the body and in patient #3, IP was seen in the left upper limb; in patient #10, IP was seen in the left face, neck, and chest, while all other cases showed pilomotor seizure symptoms over the whole body. Seizures could be accompanied by other autonomic symptoms including chills, flushing, panicking, dyspnea, or feeling hot and jumpy; however, chills was the most common concomitant symptom.

In terms of head MRI examinations, apart from seven cases with normal imaging, all others had abnormal MRI findings including atrophy, hyperintensity, or swelling in the uni‐ or bilateral hippocampi and/or amygdala. In addition, 9/16 patients showed IP during prolonged video EEG monitoring, with IP episodes usually accompanied by awareness and temporal theta‐delta rhythmic activity without generalization.

All patients with IP received immunotherapy; the treatment details and seizure frequency at the final follow‐up are shown in Table 2. All patients were given first‐line treatment. One patient (Patient #6) was treated only with intravenous gamma globulin (IVIG) administration and two patients (Patient #7 and #10) were received only corticosteroids, while the other 13 patients received IVIG plus steroids. In addition, patients received anti‐seizure medicines (ASMs), including oxcarbazepine (OXC), sodium valproate (VPA), levetiracetam (LEV), lamotrigine (LTG), and lacosamide (LCM).

**TABLE 2:** Detailed features of ictal piloerection in AE patients.

Patient	“Goose bumps” sites	Duration of IP symptoms	IP‐associated symptoms	Seizure frequency	Immunomodulant therapy	ASMs	FU since diagnosis (y)	Outcome
1	Head‐thoracic part	Seconds	No	Daily	IVIg, IV steroid, MMF	LEV, OXC	2	SF
2	Whole body	Seconds	No	Multiple/day (6–8/day)	IVIg, OS steroid	VPA, LTG	2	SF
3	Left upper limb	Seconds	No	Multiple/day (tens of times per day)	Two cycles of IVIg, IV steroid, MMF	LEV, LCM	0.5	SF
4	Whole body	Seconds	No	Once every few days	IVIg, IV steroid, MMF	VPA	1	SF
5	Whole body	Seconds	Panicking, feeling dyspnea and hot allover	Multiple/day (2–3/day, increased when having emotional stress)	IVIg, IV steroid, MMF	LCM, TPM, VPA	1.5	SF
6	Whole body	Seconds	Chills	Multiple/day (10/day, frequently presents as seizure cluster)	IVIg	LCM	1	Improvement (2–3/month)
7	Whole body	Seconds	Chills	Multiple/day (3–5/day)	IV steroid	OXC	1	Improvement (appeared after missed doses)
8	Whole body	Seconds	Cry‐face	Daily, increased when having emotional stress	IVIg, IV steroid	None	3	SF
9	Whole body	Seconds	No	Multiple/day	IVIg, IV steroid	LEV	3	SF
10	Left face, neck, and chest	Seconds	Feeling dyspnea	Multiple/day (30–40/day)	IV steroid	LEV, OXC	2	SF
11	Whole body	Seconds	Chills	Daily	IVIg, IV steroid	LEV, TPM	2	Improvement (3‐4/week)
12	Whole body	Seconds	No	Once every few days, increased when having emotional stress	Two cycles of IVIg, IV steroid	OXC, LEV, VPA	2	SF
13	Whole body	2–3 min	Feeling jumpy, nausea, and retching; occasional urinary intention	Daily	IVIg, IV steroid, MMF	LEV, TPM, LCM, OXC, VPA	3.5	Unchanged
14	Whole body	Seconds	No	Once every few days, increased when having emotional stress	IVIg, IV steroid, MMF	OXC, LEV, LCM	0.5	SF
15	Whole body	Seconds	No	Daily	IVIg, IV steroid, RTX	None	3.5	SF
16	Whole body	1 min	Flushing	Multiple/day (20–30/day)	IVIg, IV steroid	VPA, OXC	3.5	SF

Abbreviations: ASMs, anti‐seizure medications; FU, follow‐up; IP, ictal piloerection; Min, minutes; IV, intravenous; IVIg, intravenous immunoglobulins; LCM, Lacosamide; LEV, levetiracetam; LTG, lamotrigine; MMF, mycophenolate mofetil; NA, not available; OS, oral administration; OXC, oxcarbazepine; RTX, rituximab; SF, seizure‐free; T, temporal; TPM, topiramate; VPA, valproic acid; Y, years.

Apart from one patient who was lost to follow‐up (patient #16), all participants were followed up for a minimum of 6 months after discharge. The seizure frequency was significantly reduced, and 12 of 15 patients were IP seizure‐free within 3 months after discharge. Only four of the 15 continued to experience IP seizures at the final follow‐up. For patient #6, the IP frequency had decreased to 4–5/month at the 6‐month follow‐up and to 2–3/month at 12 months (final follow‐up). For patient #7, pilomotor seizures appeared after missed doses, recorded at the final follow‐up. For patient #11, the frequency had decreased to 3–4/week at 2 years (final follow‐up). And for patient #13, IP frequency remained unchanged at final follow‐up.

### 3.2 ^18^F‐FDG‐PET voxel‐wise analysis

**FIGURE 2:**
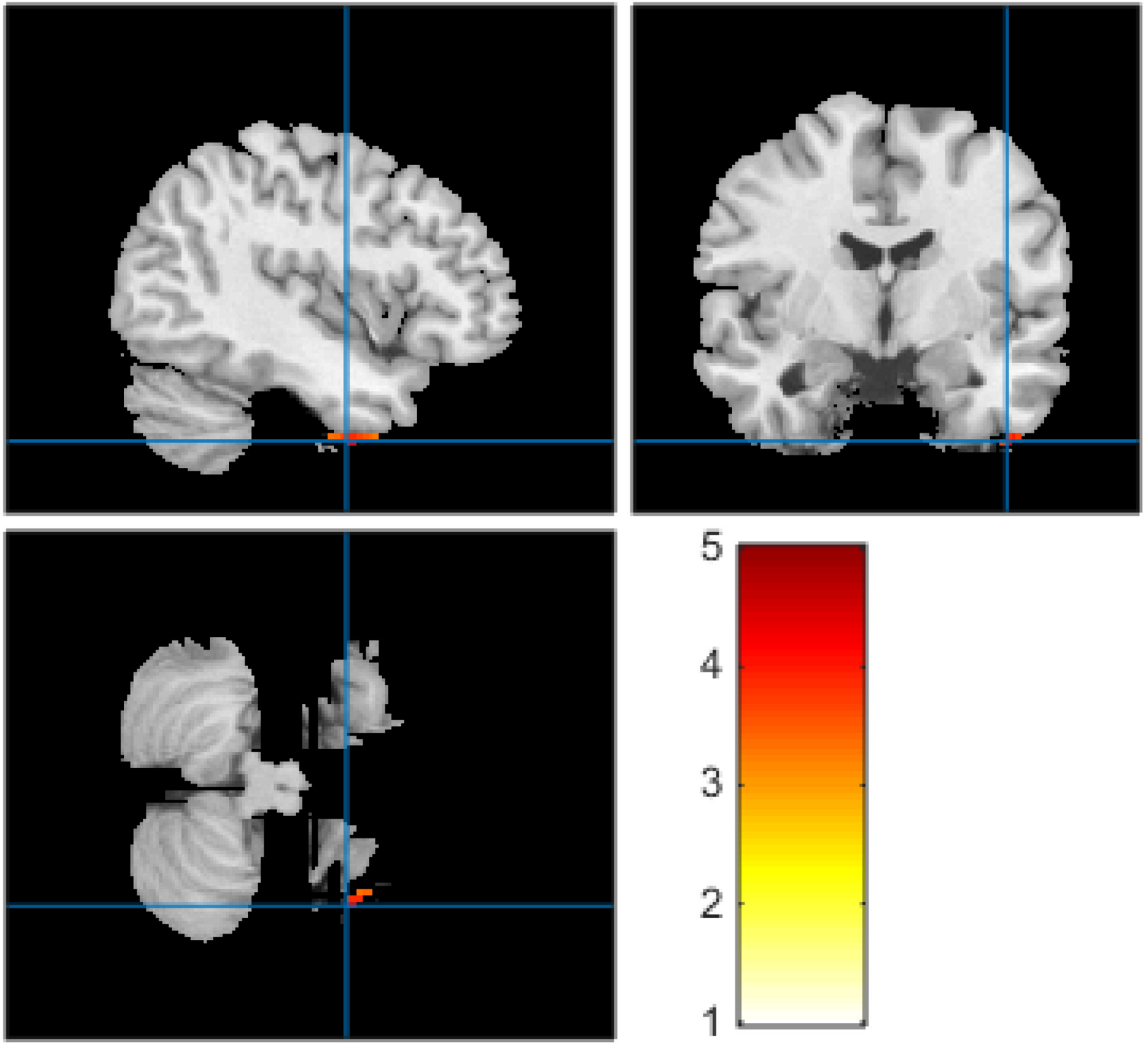
Significant cluster of brain ^18^F‐fluorodeoxyglucose positron emission tomography hypermetabolism at the group level for the patients with ictal piloerection. In comparison to patients without ictal piloerection, patients with ictal piloerection presented with significant brain hypermetabolism of the right inferior temporal gyrus (*p*‐voxel <0.001, uncorrected). Results are expressed as *T*‐score.

Of the 16 patients, two did not benefit from the PET examination and one had no available original MRI data, so they were excluded from the analyses. Statistical analysis of the whole‐brain PET imaging data showed that, relative to patients without IP, those with IP had marked clusters of hypermetabolism in the right inferior temporal gyrus (*p*‐voxel <0.001 uncorrected; *k* = 36 voxels; MNI coordinates of voxel maximum: 42, ‐4, ‐46; Figure 2). No brain areas in the group showed significant hypermetabolism. The maps of significantly hypermetabolic voxels for individual IP patients compared to those without IP are provided in Figure S1.

## 4 DISCUSSION

We reported here 16 patients with AE‐associated pilomotor seizures and suggested a specific etiological link between this type of autonomic seizure and AE. We also performed voxel‐based group comparison and demonstrated the specific symptom network associated with IP in AE.

Several studies have proposed that pilomotor seizures should be grouped as a specific multi‐seizure type associated with AE.^17^, ^18^ However, autonomic seizures rarely have IP as the main ictal symptom. The incidence of these seizures in AE is not known and IP is thought to be an uncommon symptom associated with focal epilepsy, having a predicted prevalence of 0.4%–0.65%^11^, ^19^ and 1.2% in TLE. Here, we observed an IP prevalence of 4.09% in AE patients and 12.9% in LE patients; this is consistent with extrapolations of IP prevalence of the findings on LE reported by Finke et al.^20^ and McGinty et al.^21^ (13.3% and 14%, respectively). In terms of autoantibodies, the most commonly associated antibodies were against LGI1 (68.8%) followed by NMDA (6.3%), CASPR2 (6.3%), GABAb (6.3%), GAD65 (6.3%), and combined positivity against GAD65 and mGLUR5 (6.3%). However, as IP is subtle and may not be noticed by clinicians without actively searching, it is possible that its prevalence is underestimated. Patients in our series characteristically experienced high daily frequencies of IP with durations of seconds to under 3 min, with no following loss of awareness. Besides, IP could occur on a single extremity, in the head and thorax regions, or over the entire body.

The relationships between spatial seizure organization and interictal hypermetabolism in the brain are complex, with earlier studies showing that metabolic changes are associated with the position of the seizure onset and initial propagation.^22^, ^23^, ^24^ The mechanisms underlying pilomotor seizures are obscure. Here, significant interictal hypermetabolism was seen in right inferior temporal gyrus in AE patients with IP relative to those without IP. It is thus possible that the right inferior temporal gyrus could be involved in the generation of IP. The current study appears to be first to analyze the origin of pilomotor seizures using PET which enables direct and precise identification of metabolic changes. IP is linked to bilateral or unilateral seizures generated in the temporal lobe. Rocamora et al.^11^ reported five AE cases that presented with pilomotor seizures, three of which had anti‐LGI1 encephalitis, one anti‐Hu, and one anti‐Ma2. A follow‐up study^25^ identified 15.7% cases with LGI1‐LE that had exhibited pilomotor seizures in the past, showing that the incidence is much higher in LGI1‐LE than in TLE, and thus suggesting that pilomotor seizures may be correlated with LGI1‐LE. Tényi et al.^10^ *evaluated the origin of IP seizures using combined analysis of seizure semiology, EEG, neuroimaging, and the effects of surgical treatment, finding that in 86%–94% patients, the epileptogenic area was located at the temporal lobe, with 6%–14% at extratemporal structures*. This close relationship is also confirmed by our results.

LE‐associated seizures accompanied by autoantibodies against LGI1 respond poorly to ASMs but respond well to immunotherapy.^2^ In AE patients with IP, we observed a dramatic improvement in seizures after immunotherapy. Consistent with our results to some extent, previous studies on pilomotor seizures have observed marked improved after immunotherapy.^11^, ^18^

Our study has several limitations. Firstly, the study was retrospective with a small sample size and may thus suffer from selection or recall bias. Secondly, due to the restricted sample, it is possible that lateralization of interictal hypermetabolism in the right inferior temporal gyrus may have been influenced by the greater number of bilateral or left compared to right temporal onset epilepsies in our cohort. We were thus unable to assess whether the lateralization was associated with the left, right, or bilateral temporal regions. Lastly, no patients had clinical seizures during the *PET* scan, so it is difficult to identify whole‐brain metabolic networks during ictal states. Further studies using larger sample sizes and intracranial electrode monitoring during ictal states are required.

In conclusion, focal pilomotor seizures are an uncommon symptom resulting from AE and, due to their subtle nature, are likely to be underrecognized. Our findings indicate that other AE‐associated manifestations, especially anti‐LGI1 encephalitis, should be recognized. Specifically, we observed a significant interictal hypermetabolism in the right inferior temporal gyrus associated with focal seizures accompanied by ictal piloerection.

## AUTHOR CONTRIBUTIONS

QW and YQS concepted, designed, and supervised the study. YQS and XBZ acquired the data. YQS analyzed and interpreted the data, provided statistical analysis, had full access to all of the data in the study, and are responsible for the integrity of the data and the accuracy of the data analysis. YQS drafted the manuscript, QW and LA critically revised the manuscript for important intellectual content. All authors read and approved the final manuscript.

## FUNDING INFORMATION

The study was financially supported by the National Key R&D Program of China grant (2022YFC2503800 and 2017YFC1307500), the Capital Health Research and Development of Special grants (2016‐1‐2011 and 2020‐1‐2013), the Beijing‐Tianjin‐Hebei Cooperative Basic Research Program (H2018206435), and the Beijing Natural Science Foundation (7232045 and Z200024).

## CONFLICT OF INTEREST STATEMENT

None of the authors has any conflict of interest to disclose. We confirm that we have read the Journal's position on issues involved in ethical publication and affirm that this report is consistent with those guidelines.

## Supporting information


Figure S1

## Data Availability

The data that support the findings of this study are available from the corresponding author upon reasonable request.
