# Supplementary material for: Metabolic phenotyping of pilomotor seizures in autoimmune encephalitis
Source: CNS Neurosci Ther. 2023 Mar 27;29(9):2522–9. doi: 10.1111/cns.14192 (PMC10401145; doi:10.1111/cns.14192)

**Supplementary Figures:**

Individual hypermetabolic voxels of each patient with IP in comparison to those without IP (one color for each subject: P＜0.001; left hemisphere is on the left side).


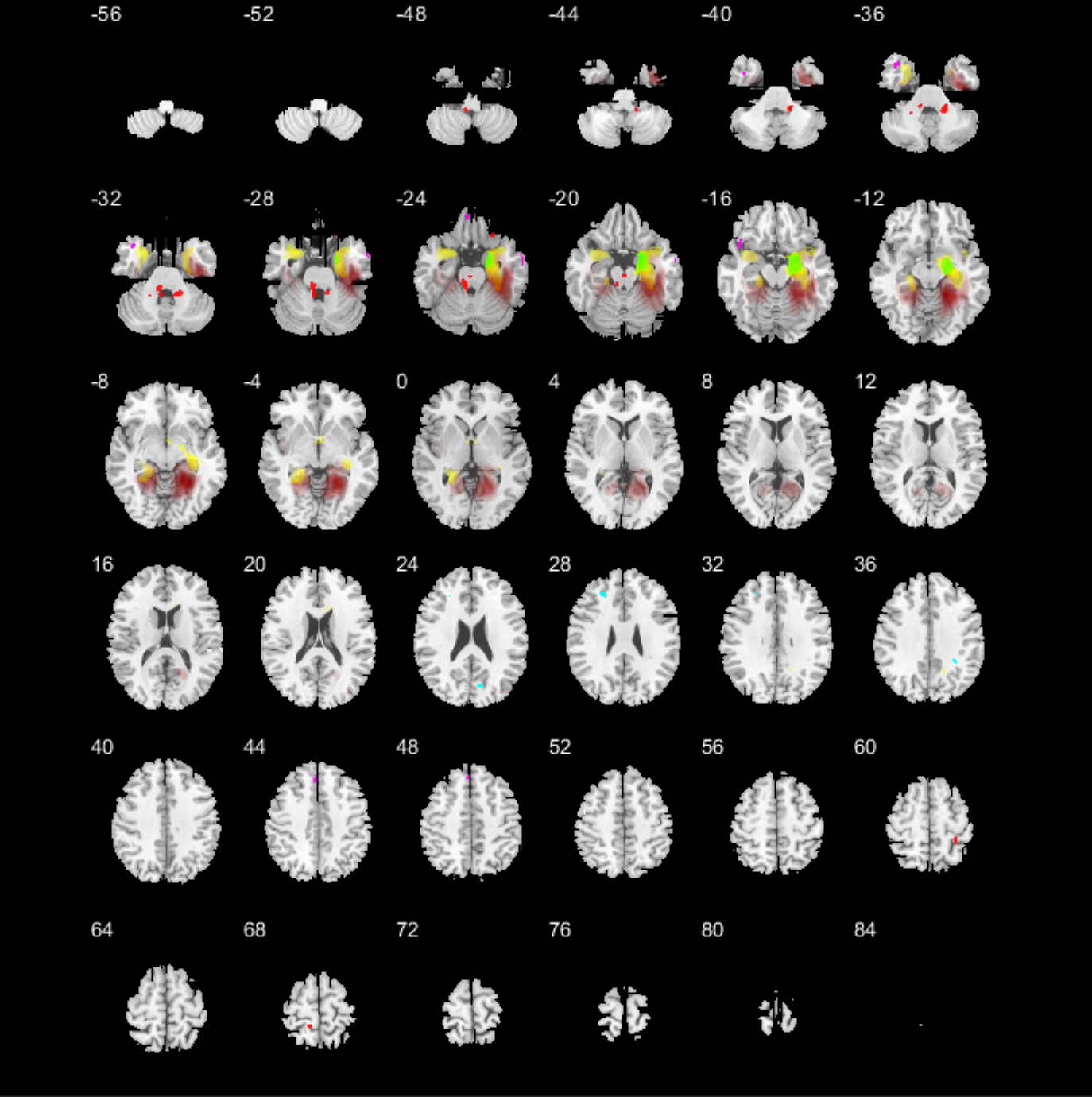


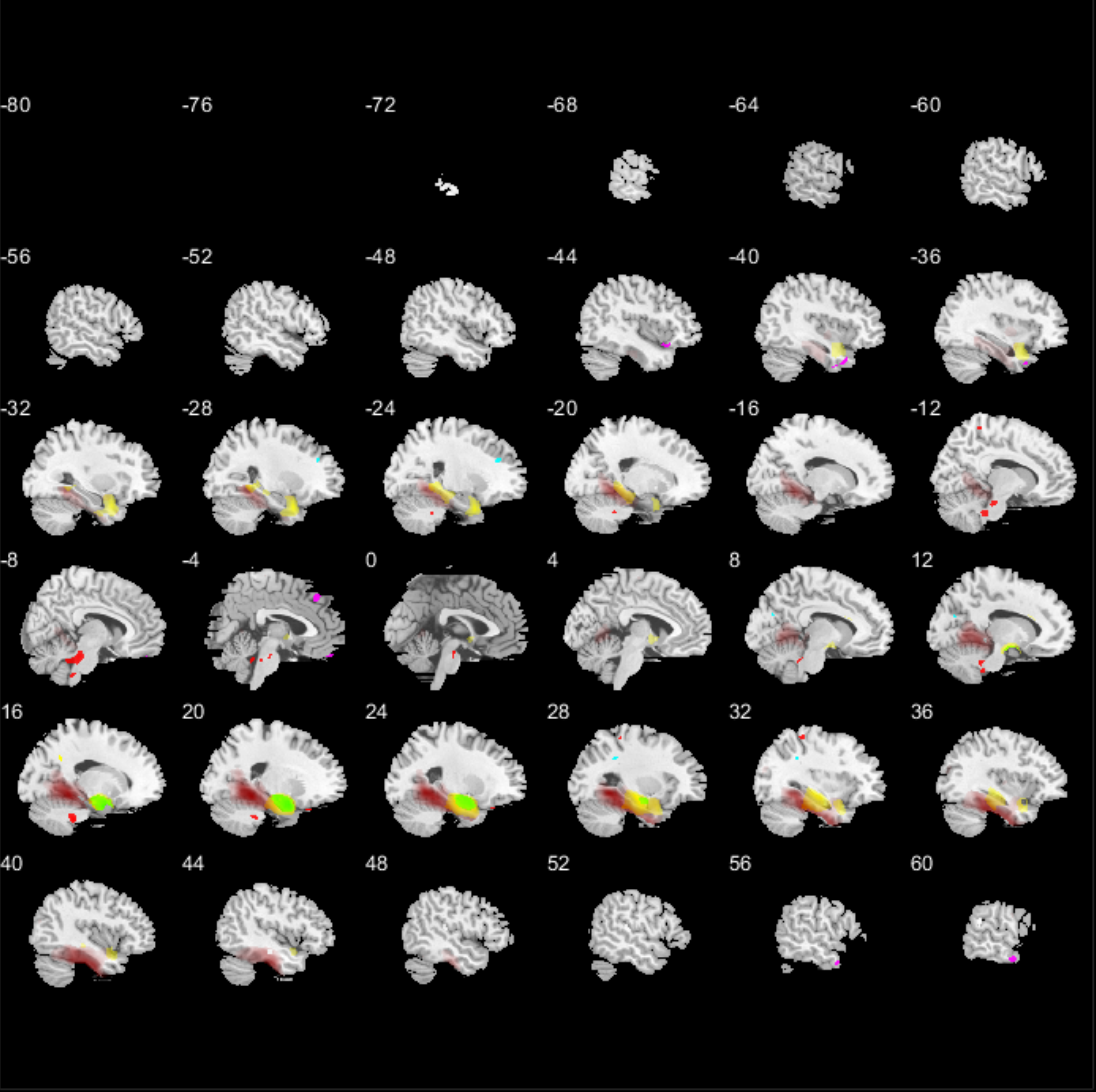

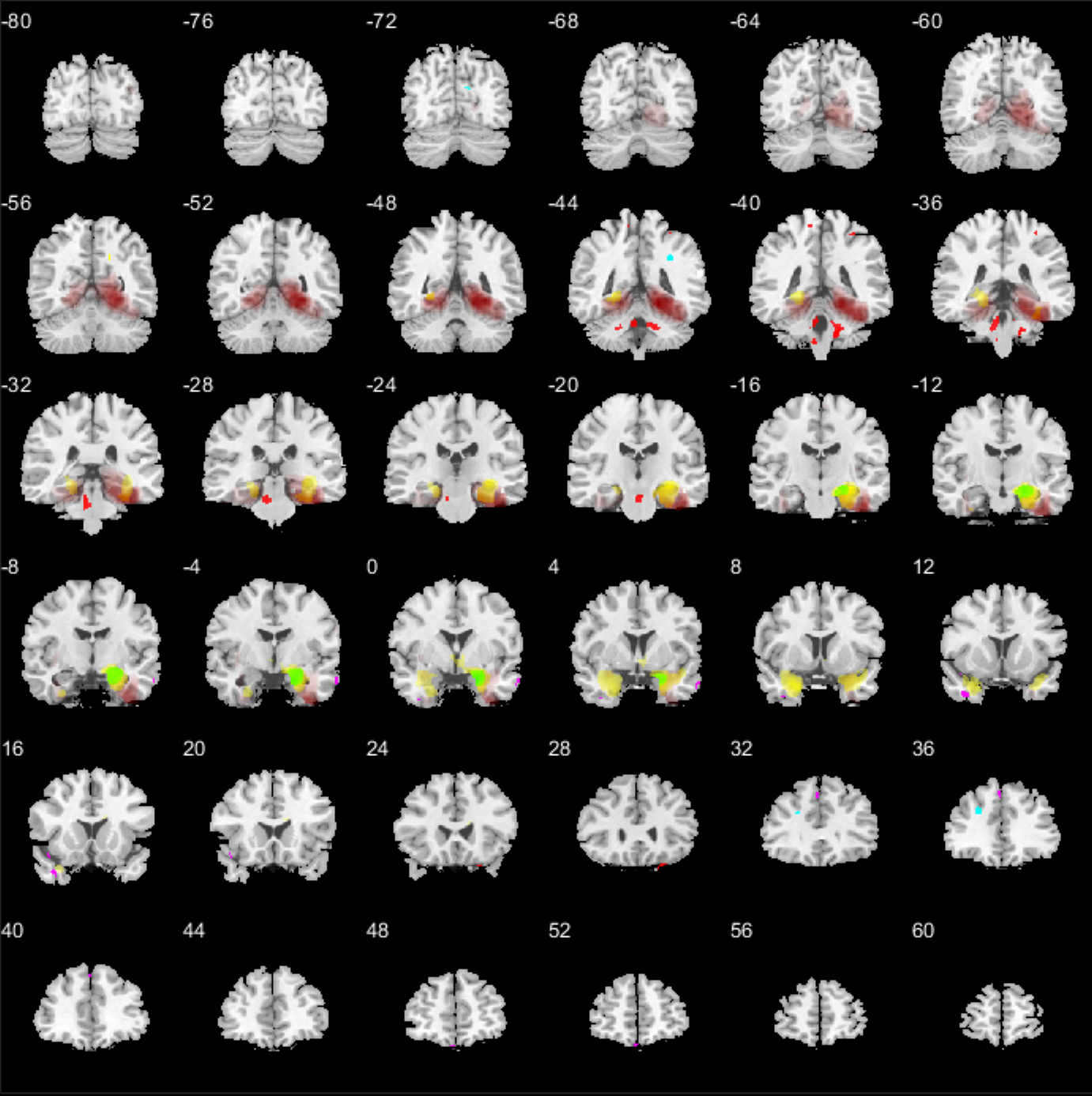

Supplement: Supplementary file 1 — Figure S1 [file CNS-29-2522-s001.docx]
